# Supplementary material for: HIVprotI: an integrated web based platform for prediction and design of HIV proteins inhibitors
Source: J Cheminform. 2018 Mar 9;10:12. doi: 10.1186/s13321-018-0266-y (PMC5845081; doi:10.1186/s13321-018-0266-y)
Supplement: Supplementary file 2 — Additional file 2. Source code of HIVProtI web server. [file 13321_2018_266_MOESM2_ESM.zip › HIVprotI_Source-code/hivprotI/weka-3-6-6/doc/weka/core/package-tree.html]

weka.core Class Hierarchy


---


|  |  |  |
| --- | --- | --- |
| **Overview** \| **Package** \| Class \| **Tree** \| **Deprecated** \| **Index** \| **Help** \| **Weka's home** | |  |
| **PREV**   **NEXT** | **FRAMES**    **NO FRAMES**     **All Classes** |  |


---


## Hierarchy For Package weka.core

**Package Hierarchies:**: All Packages

---

## Class Hierarchy

- java.lang.Object
  - weka.core.**AlgVector** (implements java.lang.Cloneable, weka.core.RevisionHandler, java.io.Serializable)- weka.core.**Attribute** (implements weka.core.Copyable, weka.core.RevisionHandler, java.io.Serializable)- weka.core.**AttributeExpression** (implements weka.core.RevisionHandler, java.io.Serializable)- weka.core.**AttributeLocator** (implements java.lang.Comparable<T>, weka.core.RevisionHandler, java.io.Serializable)
          - weka.core.**RelationalLocator**- weka.core.**StringLocator**
          - weka.core.**AttributeStats** (implements weka.core.RevisionHandler, java.io.Serializable)- weka.core.**Capabilities** (implements java.lang.Cloneable, weka.core.RevisionHandler, java.io.Serializable)- weka.core.**Check** (implements weka.core.OptionHandler, weka.core.RevisionHandler)
                - weka.core.**CheckGOE**- weka.core.**CheckOptionHandler**- weka.core.**CheckScheme**
                - weka.core.**CheckScheme.PostProcessor** (implements weka.core.RevisionHandler)- weka.core.**ClassDiscovery** (implements weka.core.RevisionHandler)- weka.core.**ClassDiscovery.StringCompare** (implements java.util.Comparator<T>, weka.core.RevisionHandler)- weka.core.**ClassloaderUtil** (implements weka.core.RevisionHandler)- weka.core.**ContingencyTables** (implements weka.core.RevisionHandler)- weka.core.**Copyright**- weka.core.**Debug** (implements weka.core.RevisionHandler, java.io.Serializable)- weka.core.**Debug.Clock** (implements weka.core.RevisionHandler, java.io.Serializable)- weka.core.**Debug.DBO** (implements weka.core.RevisionHandler, java.io.Serializable)- weka.core.**Debug.Log** (implements weka.core.RevisionHandler, java.io.Serializable)- weka.core.**Debug.SimpleLog** (implements weka.core.RevisionHandler, java.io.Serializable)- weka.core.**Debug.Timestamp** (implements weka.core.RevisionHandler, java.io.Serializable)- javax.swing.tree.DefaultMutableTreeNode (implements java.lang.Cloneable, javax.swing.tree.MutableTreeNode, java.io.Serializable)
                                          - weka.core.**Trie.TrieNode** (implements weka.core.RevisionHandler)
                                          - java.util.Dictionary<K,V>
                                            - java.util.Hashtable<K,V> (implements java.lang.Cloneable, java.util.Map<K,V>, java.io.Serializable)
                                              - java.util.Properties
                                                - weka.core.**ProtectedProperties** (implements weka.core.RevisionHandler)
                                            - weka.core.**Environment** (implements weka.core.RevisionHandler)- weka.core.**FastVector** (implements weka.core.Copyable, weka.core.RevisionHandler, java.io.Serializable)- weka.core.**FastVector.FastVectorEnumeration** (implements java.util.Enumeration<E>, weka.core.RevisionHandler)- weka.core.**FindWithCapabilities** (implements weka.core.CapabilitiesHandler, weka.core.OptionHandler, weka.core.RevisionHandler)- weka.core.**Instance** (implements weka.core.Copyable, weka.core.RevisionHandler, java.io.Serializable)
                                                      - weka.core.**SparseInstance**
                                                        - weka.core.**BinarySparseInstance**
                                                      - weka.core.**InstanceComparator** (implements java.util.Comparator<T>, weka.core.RevisionHandler, java.io.Serializable)- weka.core.**Instances** (implements weka.core.RevisionHandler, java.io.Serializable)- weka.core.**Javadoc** (implements weka.core.OptionHandler, weka.core.RevisionHandler)
                                                            - weka.core.**AllJavadoc**- weka.core.**GlobalInfoJavadoc**- weka.core.**OptionHandlerJavadoc**- weka.core.**TechnicalInformationHandlerJavadoc**
                                                            - weka.core.**Jython** (implements weka.core.RevisionHandler, java.io.Serializable)- weka.core.**ListOptions** (implements weka.core.OptionHandler, weka.core.RevisionHandler)- weka.core.**MathematicalExpression** (implements weka.core.RevisionHandler)- weka.core.**Matrix** (implements java.lang.Cloneable, weka.core.RevisionHandler, java.io.Serializable)- weka.core.**Memory** (implements weka.core.RevisionHandler)- weka.core.**NormalizableDistance** (implements weka.core.DistanceFunction, weka.core.OptionHandler, weka.core.RevisionHandler, java.io.Serializable)
                                                                        - weka.core.**AbstractStringDistanceFunction**
                                                                          - weka.core.**EditDistance**
                                                                          - weka.core.**ChebyshevDistance** (implements weka.core.TechnicalInformationHandler)- weka.core.**EuclideanDistance** (implements java.lang.Cloneable, weka.core.TechnicalInformationHandler)- weka.core.**ManhattanDistance** (implements weka.core.TechnicalInformationHandler)
                                                                        - weka.core.**Optimization** (implements weka.core.RevisionHandler, weka.core.TechnicalInformationHandler)- weka.core.**Option** (implements weka.core.RevisionHandler)- java.io.OutputStream (implements java.io.Closeable, java.io.Flushable)
                                                                              - java.io.FilterOutputStream
                                                                                - java.io.PrintStream (implements java.lang.Appendable, java.io.Closeable)
                                                                                  - weka.core.**Tee** (implements weka.core.RevisionHandler)
                                                                              - weka.core.**PropertyPath** (implements weka.core.RevisionHandler)- weka.core.**PropertyPath.Path** (implements weka.core.RevisionHandler)- weka.core.**PropertyPath.PathElement** (implements java.lang.Cloneable, weka.core.RevisionHandler)- weka.core.**Queue** (implements weka.core.RevisionHandler, java.io.Serializable)- java.util.Random (implements java.io.Serializable)
                                                                                        - weka.core.**Debug.Random** (implements weka.core.RevisionHandler, java.io.Serializable)- weka.core.**RandomVariates** (implements weka.core.RevisionHandler)
                                                                                        - weka.core.**Range** (implements weka.core.RevisionHandler, java.io.Serializable)- weka.core.**RevisionUtils**- weka.core.**SelectedTag** (implements weka.core.RevisionHandler)- weka.core.**SerializationHelper** (implements weka.core.RevisionHandler)- weka.core.**SerializedObject** (implements weka.core.RevisionHandler, java.io.Serializable)- weka.core.**SingleIndex** (implements weka.core.RevisionHandler, java.io.Serializable)- weka.core.**SpecialFunctions** (implements weka.core.RevisionHandler)- weka.core.**Statistics** (implements weka.core.RevisionHandler)- weka.core.**Stopwords** (implements weka.core.RevisionHandler)- weka.core.**SystemInfo** (implements weka.core.RevisionHandler)- weka.core.**Tag** (implements weka.core.RevisionHandler, java.io.Serializable)- weka.core.**TechnicalInformation** (implements weka.core.RevisionHandler)- weka.core.**TestInstances** (implements java.lang.Cloneable, weka.core.OptionHandler, weka.core.RevisionHandler, java.io.Serializable)- java.lang.Throwable (implements java.io.Serializable)
                                                                                                                    - java.lang.Exception
                                                                                                                      - java.lang.RuntimeException
                                                                                                                        - weka.core.**UnassignedClassException**- weka.core.**UnassignedDatasetException**
                                                                                                                        - weka.core.**WekaException**
                                                                                                                          - weka.core.**NoSupportForMissingValuesException**- weka.core.**UnsupportedAttributeTypeException**- weka.core.**UnsupportedClassTypeException**
                                                                                                                    - weka.core.**Trie** (implements java.lang.Cloneable, java.util.Collection<E>, weka.core.RevisionHandler, java.io.Serializable)- weka.core.**Trie.TrieIterator** (implements java.util.Iterator<E>, weka.core.RevisionHandler)- weka.core.**Utils** (implements weka.core.RevisionHandler)- weka.core.**Version** (implements java.lang.Comparable<T>, weka.core.RevisionHandler)

## Interface Hierarchy

- weka.core.**AdditionalMeasureProducer**- weka.core.**CapabilitiesHandler**
    - weka.core.**MultiInstanceCapabilitiesHandler**
    - weka.core.**Copyable**- weka.core.**Drawable**- weka.core.**EnvironmentHandler**- weka.core.**JythonObject**
            - weka.core.**JythonSerializableObject** (also extends java.io.Serializable)
            - weka.core.**Matchable**- weka.core.**OptionHandler**
                - weka.core.**DistanceFunction**
                - weka.core.**Randomizable**- weka.core.**RevisionHandler**- java.io.Serializable
                      - weka.core.**JythonSerializableObject** (also extends weka.core.JythonObject)
                      - weka.core.**Summarizable**- weka.core.**TechnicalInformationHandler**- weka.core.**Undoable**- weka.core.**WeightedInstancesHandler**

## Enum Hierarchy

- java.lang.Object
  - java.lang.Enum<E> (implements java.lang.Comparable<T>, java.io.Serializable)
    - weka.core.**TechnicalInformation.Type**- weka.core.**TechnicalInformation.Field**- weka.core.**RevisionUtils.Type**- weka.core.**Capabilities.Capability**

---


|  |  |  |
| --- | --- | --- |
| **Overview** \| **Package** \| Class \| **Tree** \| **Deprecated** \| **Index** \| **Help** \| **Weka's home** | |  |
| **PREV**   **NEXT** | **FRAMES**    **NO FRAMES**     **All Classes** |  |


---
